# Adenosine A_2A_ Receptor Up-Regulates Retinal Wave Frequency via Starburst Amacrine Cells in the Developing Rat Retina

**DOI:** 10.1371/journal.pone.0095090

**Published:** 2014-04-28

**Authors:** Pin-Chien Huang, Yu-Tien Hsiao, Shao-Yen Kao, Ching-Feng Chen, Yu-Chieh Chen, Chung-Wei Chiang, Chien-fei Lee, Juu-Chin Lu, Yijuang Chern, Chih-Tien Wang

**Affiliations:** 1 Institute of Molecular and Cellular Biology, National Taiwan University, Taipei, Taiwan; 2 Department of Life Science, National Taiwan University, Taipei, Taiwan; 3 Neurobiology and Cognitive Science Center, National Taiwan University, Taipei, Taiwan; 4 Genome and Systems Biology Degree Program, National Taiwan University, Taipei, Taiwan; 5 Department of Physiology and Pharmacology, College of Medicine, Chang Gung University, Tao-Yuan, Taiwan; 6 Institute of Biomedical Sciences, Academia Sinica, Taipei, Taiwan; Universidade Federal do ABC, Brazil

## Abstract

**Background:**

Developing retinas display retinal waves, the patterned spontaneous activity essential for circuit refinement. During the first postnatal week in rodents, retinal waves are mediated by synaptic transmission between starburst amacrine cells (SACs) and retinal ganglion cells (RGCs). The neuromodulator adenosine is essential for the generation of retinal waves. However, the cellular basis underlying adenosine's regulation of retinal waves remains elusive. Here, we investigated whether and how the adenosine A_2A_ receptor (A_2A_R) regulates retinal waves and whether A_2A_R regulation of retinal waves acts via presynaptic SACs.

**Methodology/Principal Findings:**

We showed that A_2A_R was expressed in the inner plexiform layer and ganglion cell layer of the developing rat retina. Knockdown of A_2A_R decreased the frequency of spontaneous Ca^2+^ transients, suggesting that endogenous A_2A_R may up-regulate wave frequency. To investigate whether A_2A_R acts via presynaptic SACs, we targeted gene expression to SACs by the metabotropic glutamate receptor type II promoter. Ca^2+^ transient frequency was increased by expressing wild-type A_2A_R (A_2A_R-WT) in SACs, suggesting that A_2A_R may up-regulate retinal waves via presynaptic SACs. Subsequent patch-clamp recordings on RGCs revealed that presynaptic A_2A_R-WT increased the frequency of wave-associated postsynaptic currents (PSCs) or depolarizations compared to the control, without changing the RGC's excitability, membrane potentials, or PSC charge. These findings suggest that presynaptic A_2A_R may not affect the membrane properties of postsynaptic RGCs. In contrast, by expressing the C-terminal truncated A_2A_R mutant (A_2A_R-ΔC) in SACs, the wave frequency was reduced compared to the A_2A_R-WT, but was similar to the control, suggesting that the full-length A_2A_R in SACs is required for A_2A_R up-regulation of retinal waves.

**Conclusions/Significance:**

A_2A_R up-regulates the frequency of retinal waves via presynaptic SACs, requiring its full-length protein structure. Thus, by coupling with the downstream intracellular signaling, A_2A_R may have a great capacity to modulate patterned spontaneous activity during neural circuit refinement.

## Introduction

During a critical period in the developing retina, immature retinal ganglion cells (RGCs) spontaneously fire periodic bursts of action potentials that propagate across the retina, encompassing hundreds to thousands of cells [1], [2]. These “retinal waves” occur prior to visual experience, with a periodicity on the order of minutes [1], [2]. Three different stages of retinal waves have been classified in the developing mammalian retina according to their initiation mechanisms [2], [3], [4]; the stage-II waves have been shown to be critical for the refinement of retinal projections to central brain targets [5], [6], [7], [8], [9], [10], [11], [12]. The stage-II waves (during postnatal days P0-P9 in rats) [13], [14] are mediated by a subset of starburst amacrine cells (SACs) releasing acetylcholine (ACh) and γ-aminobutyric acid (GABA) (inducing neuronal depolarization during this period [14], [15]) onto neighboring SACs and RGCs [16], [17], [18], [19]. Thus, periodic, correlated depolarizations and Ca^2+^ oscillations propagate across the RGC layer in a wave-like manner [2], [16], [19].

The neuromodulator adenosine is essential for the generation of retinal waves [3], [4], [14], [20], [21]. The elimination of extracellular adenosine by adenosine deaminase blocks the generation of retinal waves [20]. Previous studies found that adenosine exerted its effects by activating four distinct types of G-protein-coupled receptors (GPCRs) classified as A_1_R, A_2A_R, A_2B_R, and A_3_R [22], [23]. The frequency of retinal waves was increased by activation of the adenosine A2 receptor (A_2_R) by a general agonist, 5′-N-ethylcarboxamido adenosine (NECA), suggesting that A_2_R activation was a positive regulator of retinal wave periodicity [20]. Furthermore, pharmacology experiments indicated that wave frequency was not altered by blocking specific receptor subtypes, including A_1_R, A_2B_R, and A_3_R [14]. However, blocking the adenosine A_2A_ receptor (A_2A_R) with a selective antagonist (ZM 241385) increased the frequency of retinal waves [14], inconsistent with the results from NECA application [20]. Moreover, a general adenosine receptor antagonist, aminophylline, which blocks waves [3], [21], was later found to act as a GABA_A_R agonist mediating tonic activation that can alter the correlation structure of stage-II waves [14], [15]. These results suggest that revisiting the pharmacological results is necessary to verify the role of adenosine signaling in regulating retinal waves.

Although the A_2A_R has been implicated in regulating retinal waves [14], [20], it is unclear whether A_2A_R regulates stage-II waves in a positive or negative manner [14], [20], or whether its regulation acts via presynaptic SACs or postsynaptic RGCs. Of particular interest, compared to other adenosine receptor subtypes, A_2A_R confers a relatively long intracellular C-terminus that is highly conserved for all cloned species [24]. Several A_2A_R-interacting proteins, which bind to the C-terminus of A_2A_R and regulate the cyclic AMP (cAMP)-dependent and -independent signaling pathways upon activation, were reported earlier [25], [26]. However, it remains completely unknown whether the long C-terminus of A_2A_R is required for the regulation of retinal waves.

Here, we combined molecular perturbation (knockdown or cell type-specific expression), live Ca^2+^ imaging, and whole-cell patch-clamp recordings to investigate how A_2A_R regulates retinal waves, if A_2A_R regulation of retinal waves acts via presynaptic SACs, and whether full-length A_2A_R is required for regulation of stage-II waves during visual circuit refinement.

## Results

### A_2A_R is expressed in the IPL and GCL of the developing rat retina

**Figure 1:**
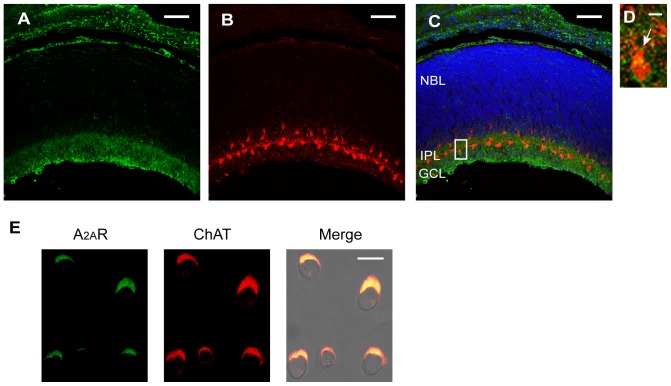
A_2A_R is expressed in rat postnatal IPL and GCL. A–B. Immunofluorescence staining of (A) the adenosine A_2A_ receptor (A_2A_R) and (B) choline acetyltransferase (ChAT) in retinal cross-sections from P2 rats. C. The merged image of the A_2A_R (green) and ChAT (red) staining. The cell nuclei were stained with DAPI (blue). NBL, neuroblast layer; IPL, inner plexiform layer; GCL, ganglion cell layer. D. The high magnification of the merged image in the box of C. The arrow indicated a starburst amacrine cell (SAC). E. Immunofluorescence staining of A_2A_R (green) and ChAT (red) in single SACs dissociated from the P2 rat retinas. *Right*, the merged image under the bright field. The colocalization signals were shown in yellow. Scale bars for A–C, 50 µm. Scale bar for D, 5 µm. Scale bar for E, 7.5 µm.

To determine whether A_2A_R plays a role in regulating retinal waves in rats, we first examined whether A_2A_R is expressed in the developing rat retina exhibiting stage-II waves. Because stage-II waves are mediated by synaptic transmission between SACs and RGCs, we labeled SACs using a marker of cholinergic neurons (choline acetyltransferase, ChAT). Double immunostaining for A_2A_R (Fig. 1A) and ChAT (Fig. 1B) was applied to P2 rat retinal cross-sections. Further DAPI staining on nuclei allowed us to distinguish the ganglion cell layer (GCL, the layer containing RGCs and displaced SACs), the inner plexiform layer (IPL, the layer containing SAC-RGC synapses), and the neuroblast layer (NBL) (Fig. 1C). We found that A_2A_R immunoreactivity was localized to the IPL and the GCL but not the NBL or other regions of the postnatal rat retina (Fig. 1A and C), consistent with A_2A_R expression in the GCL and amacrine cell layer in the developing ferret retina [20]. Other A_2A_R immunoreactivity was found in non-neuronal cell types, such as the retinal pigment epithelium and choroid (Fig. 1A and C), consistent with the results from localization of A_2A_R mRNA in the rat eye [27]. These results suggest that A_2A_R is present in the rat inner retina, which has been shown to participate in stage-II waves [14]. Moreover, the high-magnification image showed that A_2A_R immunoreactivity localized to the wave-generating cells, SACs, in the retinal cross-section (Fig. 1D). Further immunostaining in the dissociated SACs confirmed the colocalization of A_2A_R and ChAT immunoreactivities (Fig. 1E), indicating that A_2A_R is expressed in SACs. These results suggest that A_2A_R may play a role in modulating stage-II waves in the developing rat retina.

### Knockdown of endogenous A_2A_R decreases Ca^2+^ transient frequency

**Figure 2:**
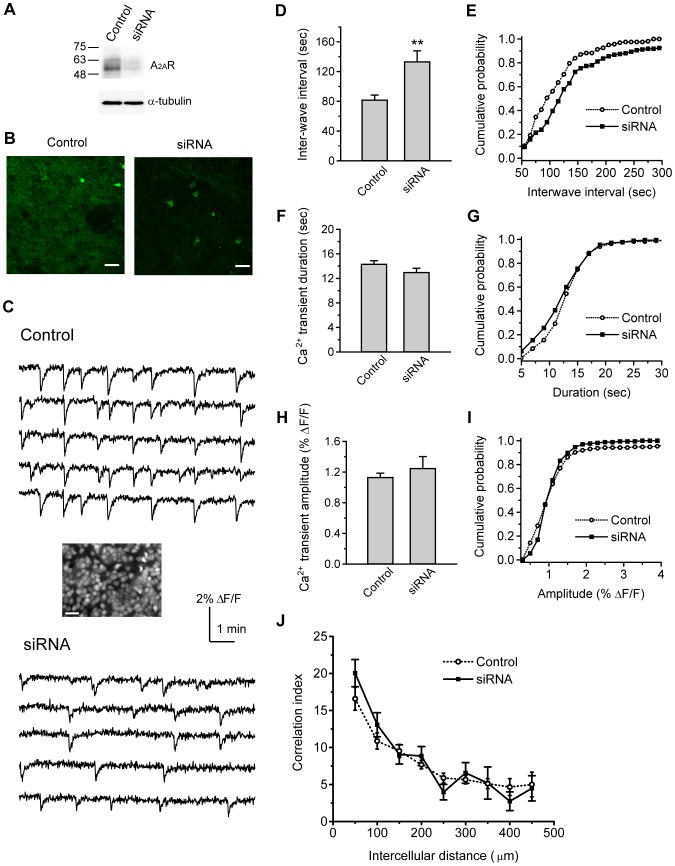
Knockdown of endogenous A_2A_R reduces Ca^2+^ transient frequency in the developing rat retina. A. Knockdown of A_2A_R-siRNA in PC12 cells. Cells transfected with pSuper-hrGFP (Control) or pSuper-hrGFP carrying A_2A_R-siRNA (siRNA) were subjected to Western blot analysis with antibodies indicated on the right (A_2A_R or α-tubulin). B. Knockdown of endogenous A_2A_R in postnatal rat retinas. Whole-mount retinas from P2 rats were transfected with control vector (Control) or A_2A_R-siRNA (siRNA). Seventy-two hr post transfection, the retinas were immunolabeled with A_2A_R antibody (green). Scale bars, 25 µm. C. Representative traces of fluorescence changes over time showed spontaneous, correlated Ca^2+^ transients in the nearby cells on the RGC layer from the retinas transfected with control vector (Control) or A_2A_R-siRNA (siRNA). Inset, The RGC layer was labeled with the Ca^2+^ indicator fura-2 to measure the wave-associated Ca^2+^ transients after transfection. Scale bar, 20 µm. D. Summary of the inter-wave interval for correlated Ca^2+^ transients after A_2A_R knockdown. ** *p*<0.01; two-tailed Student's unpaired *t*-test. E. Distributions of cumulative probability for the inter-wave interval from individual cells. *p*<0.01; Kolmogorov-Smirnov test. F. Summary of Ca^2+^ transient duration after A_2A_R knockdown. *p* = 0.14; two-tailed Student's unpaired *t*-test. G. Distributions of cumulative probability for Ca^2+^ transient duration from individual cells. *p* = 0.07; Kolmogorov-Smirnov test. H. Summary of Ca^2+^ transient amplitude after A_2A_R knockdown. *p* = 0.49; two-tailed Student's unpaired *t*-test. I. Distributions of cumulative probability for Ca^2+^ transient amplitude from individual cells. *p* = 0.09; Kolmogorov-Smirnov test. J. Pairwise correlation after A_2A_R knockdown. *p*>0.05 for each given distance; two-tailed Student's unpaired *t*-test. For D–J, data were obtained from 170–180 cells, 11 transfected retinas, and 4 pups.

To determine the role of A_2A_R in modulating stage-II waves, we expressed the siRNA against A_2A_R (A_2A_R-siRNA) in retinal explants using the electroporation strategy established in our previous study [28]. The specificity of the A_2A_R-siRNA was first examined in the cell line by Western blot analysis (Fig. 2A), and the knockdown efficiency by the siRNA was quantified as 0.23±0.07 compared to control (N = 3). With this A_2A_R-siRNA, the specificity of A_2A_R antibody was verified by immunofluorescence staining in the whole-mount retinal explants (Fig. 2B). Under the same staining and imaging condition in retinal explants, we found that the A_2A_R-siRNA reduced the A_2A_R immunoreactivity by half compared to the control (fluorescence intensity: 0.55±0.03 normalized to control, N = 12 retinas).

To determine the effects of endogenous A_2A_R on spontaneous Ca^2+^ transients, live Ca^2+^ imaging was conducted in the retinal explants after knockdown of A_2A_R (Fig. 2C). Spontaneous, correlated Ca^2+^ transients in individual cells revealed stage-II waves in the RGC layer of transfected explants. To eliminate the variance across cells or retinas, the mean inter-wave interval was calculated from a number of transfected retinas for the measurement of wave frequency. We found that knockdown of A_2A_R increased the mean interval of Ca^2+^ transients by approximately 2-fold compared to the control (*p*<0.01, Fig. 2D), suggesting that knockdown of A_2A_R decreased the wave frequency. Moreover, we constructed the cumulative probability for the inter-wave interval from all recorded cells. The curve of cumulative probability for the inter-wave interval was significantly right-shifted after A_2A_R knockdown (*p*<0.01, Fig. 2E), suggesting that the majority of cells after A_2A_R knockdown displayed the longer interval compared to control. These results suggest that depletion of endogenous A_2A_R reduced wave frequency. Thus, endogenous A_2A_R may up-regulate wave frequency in the developing rat retina.

A spontaneous Ca^2+^ transient in single cells lasts for tens of seconds and this unique temporal pattern is important for activation of the downstream cAMP/protein kinase A (PKA) signaling [13]. To examine whether knockdown of A_2A_R alters the wave pattern, we measured the duration and amplitude of individual Ca^2+^ transients, using the criteria set by a program developed in our previous study [28]. We found that knockdown of A_2A_R did not significantly change the duration or amplitude of Ca^2+^ transients compared to the control (Fig. 2F and H). In addition, the curve of cumulative probability for Ca^2+^ transient duration or amplitude was not shifted by knockdown of A_2A_R (Fig. 2G and I). Thus, endogenous A_2A_R may not play a role in regulating the wave pattern.

To determine whether knockdown of A_2A_R affects the spatial properties of stage-II waves, the pairwise correlation index (C.I.) [28], [29], [30] was computed and plotted against the intercellular distance (Fig. 2J). In both the control and A_2A_R-siRNA, the C.I. values were decreased as a function of distance in a similar fashion, suggesting that the propagating waves were persistent after knockdown of A_2A_R. Moreover, the C.I. values at any given distance were not significantly different when comparing the control and A_2A_R knockdown. Thus, endogenous A_2A_R may not play a role in regulating the spatial correlation structure of stage-II waves.

### SAC-specific expression of wild-type A_2A_R increases Ca^2+^ transient frequency

Knockdown of A_2A_R decreases Ca^2+^ transient frequency without altering the amplitude or spatial correlation of Ca^2+^ transients, implying that the effects are presynaptic. SACs are wave-generating cells that set the rhythmic periodicity of stage-II waves [18], [19]. Thus, it is likely that endogenous A_2A_R may act via SACs to up-regulate the frequency of retinal waves. To test this hypothesis, we targeted expression of A_2A_R or its mutant to SACs with the metabotropic glutamate receptor type II (mGluR2) promoter [14], [19], [31], [32]. Our previous study has also shown that the mGluR2 promoter can drive SAC-specific expression in retinal explants with ∼84% of the cells targeted to SACs compared to 8% targeted to SACs with the CMV promoter [28]. Moreover, using a horizontal electroporation configuration, gene expression driven by the mGluR2 promoter can achieve high transfection efficiency (∼50%) that is sufficient to modulate the molecular machinery in SACs and further alters the dynamics of retinal waves [28]. Hence, in the following experiments, we utilized the mGluR2 promoter to target expression of A_2A_R or its mutant to SACs. Similar to the previous study [28], we confirmed that transfected retinal explants reliably demonstrated EGFP fluorescence in relatively small cells (∼5 µm), and the EGFP expression pattern was essentially the same among all transfection groups, suggesting that transfection efficiency was comparable in all groups.

To check the A_2A_R expression under the control of the mGluR2 promoter, we performed immunostaining in the whole-amount retinas transfected with control vector (Figure S1-A and D), wild-type A_2A_R (A_2A_R-WT) (Figure S1-B and E), or the C-terminal truncated A_2A_R mutant (A_2A_R-ΔC) (Figure S1-C and F). The A_2A_R immunoreactivity was distributed across the IPL in these transfected groups, partially localized to SACs. To determine whether the mGluR2 promoter can achieve SAC-specific overexpression of A_2A_R or its mutant, double immunofluorescence staining was further performed in dissociated SACs. Our results showed that, compared to the control, A_2A_R immunoreactivity was significantly higher in SACs by mGluR2 promoter-driven expression of A_2A_R-WT or A_2A_R-ΔC (*p*<0.01, Figure S1-G and H), suggesting that the mGluR2 promoter can overexpress A_2A_R or its mutant in SACs. In addition, the expression pattern of A_2A_R immunoreactivity was comparable in the control, A_2A_R-WT, or A_2A_R-ΔC (Figure S1-G), suggesting that the subcellular localization may not be altered by overexpression of these receptors.

**Figure 3:**
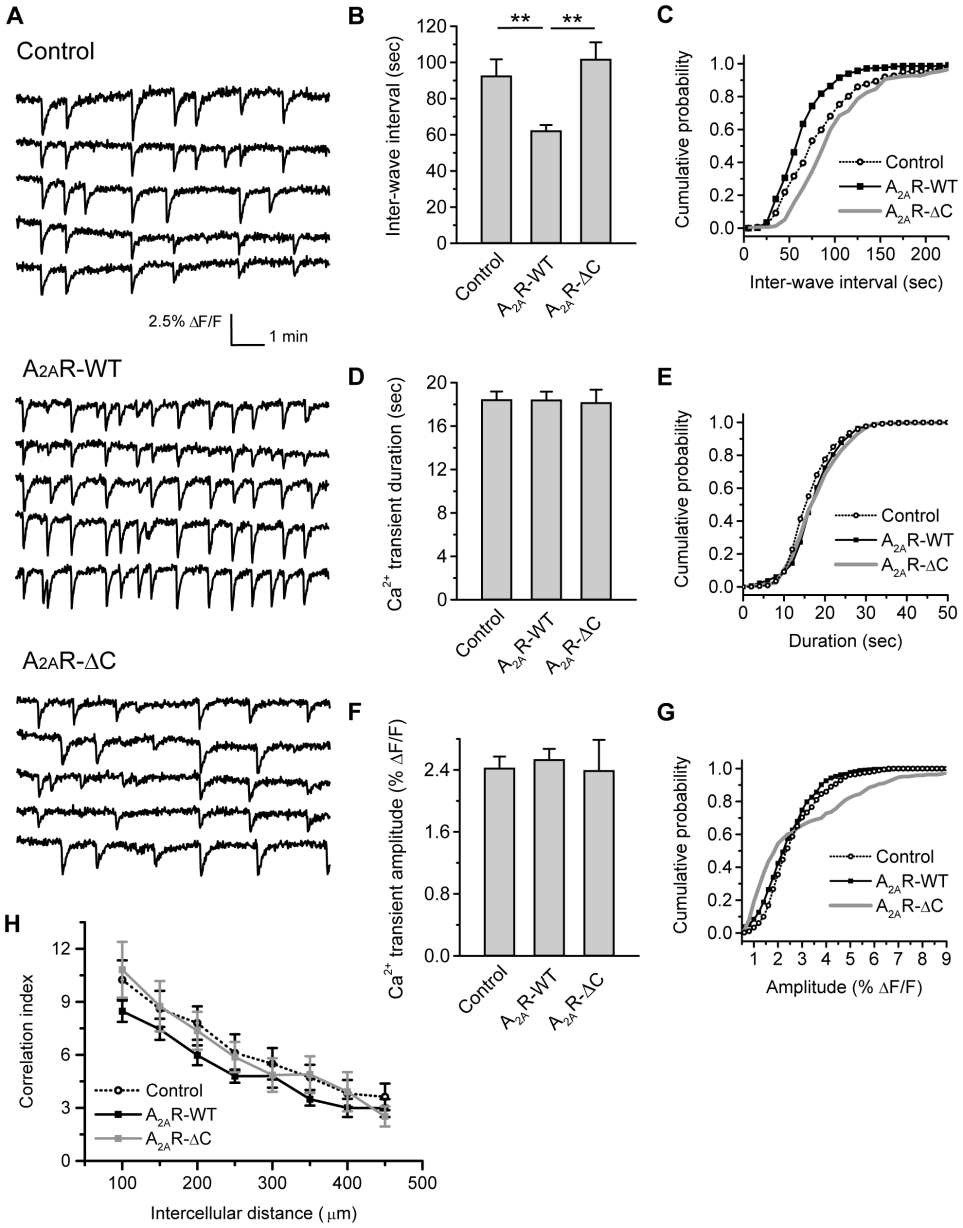
A_2A_R-WT, but not A_2A_R-ΔC, increases Ca^2+^ transient frequency from SAC. A. Representative traces of spontaneous Ca^2+^ transients in the nearby cells of the RGC layer. Retinas were transfected with pmGluR2-IRES2EGFP (Control), pmGluR2-IRES2EGFP-wild-type A_2A_R (A_2A_R-WT), or pmGluR2-IRES2EGFP-C-terminal-deletion mutant of A_2A_R (A_2A_R-ΔC) for SAC-specific expression. B. Summary of the inter-wave interval for correlated Ca^2+^ transients. ** *p*<0.01; Kruskal-Wallis method followed by a *post*-*hoc* Dunn test. C. Distributions of cumulative probability for the inter-wave interval from individual cells. *p*<0.001 for Control vs. A_2A_R-WT, A_2A_R-WT vs. A_2A_R-ΔC, and Control vs. A_2A_R-ΔC; Kolmogorov-Smirnov test. D. Summary of Ca^2+^ transient duration. *p* = 0.88; Kruskal-Wallis method with a *post*-*hoc* Dunn test. E. Distributions of cumulative probability for Ca^2+^ transient duration from individual cells. *p*<0.001 for Control vs. A_2A_R-WT, *p* = 0.41 for A_2A_R-WT vs. A_2A_R-ΔC, and *p*<0.01 for Control vs. A_2A_R-ΔC; Kolmogorov-Smirnov test. F. Summary of Ca^2+^ transient amplitude. *p* = 0.30; Kruskal-Wallis method with a *post*-*hoc* Dunn test. G. Distributions of cumulative probability for Ca^2+^ transient amplitude from individual cells. *p*<0.05 for Control vs. A_2A_R-WT, *p*<0.001 for A_2A_R-WT vs. A_2A_R-ΔC, and *p*<0.001 for Control vs. A_2A_R-ΔC; Kolmogorov-Smirnov test. H. Pairwise correlation. *p*>0.05 for each given distance; One-way ANOVA with a *post*-*hoc* Student-Newman-Keuls test. For B–H, data were obtained from 250–420 cells, 15–27 transfected retinas, and 4–9 pups.

To determine whether A_2A_R expression in presynaptic SACs up-regulates retinal waves, we examined spontaneous Ca^2+^ transients in retinal explants expressing A_2A_R under the control of the mGluR2 promoter (Fig. 3). SAC-specific expression of A_2A_R-WT significantly decreased the interval of spontaneous Ca^2+^ transients compared to the control (*p*<0.01, Fig. 3A and B). The curve of cumulative probability for the inter-wave interval was left-shifted by SAC-specific expression of the A_2A_R-WT (*p*<0.001, Fig. 3C), suggesting that the majority of cells display Ca^2+^ transients more frequently compared to the control. By contrast, SAC-specific expression of the A_2A_R-WT did not alter the mean duration (Fig. 3D), mean amplitude (Fig. 3F), or spatial correlation (Fig. 3H) of spontaneous Ca^2+^ transients. Although significant differences were obtained in the cumulative probability curves for duration or amplitude (Fig. 3E and G), these effects from single cells were diminished by taking the averages across a number of cells and retinas (Fig. 3D and F). Hence, SAC-specific expression of the A_2A_R-WT had relatively minor effects on Ca^2+^ transient duration or amplitude. In addition to the mGluR2 promoter, we also examined overexpression of A_2A_R under the control of the CMV promoter, which achieves efficient overexpression in retinal explants but fails to specifically target to SACs [28]. However, by expressing A_2A_R with the CMV promoter, we detected no significant changes in terms of the Ca^2+^ transient characteristics, such as the inter-wave interval, duration, or amplitude, compared to the control (Table S1). These results suggest that the presynaptic SACs may serve as the functional locus for A_2A_R up-regulation of wave frequency.

### Ca^2+^ transient frequency is not altered by SAC-specific expression of the C-terminal truncated A_2A_R mutant

A_2A_R confers a relatively long intracellular C-terminus compared to other adenosine receptor subtypes [24]. Previous studies have shown that this long C-terminus mediates A_2A_R's intracellular signaling upon receptor activation [25], [26], [33]. To test whether A_2A_R up-regulation of retinal waves involves the intracellular signaling, we expressed the C-terminal truncated A_2A_R mutant (A_2A_R-ΔC) in SACs. We found that compared to the control, SAC-specific expression of the A_2A_R-ΔC did not significantly alter the mean inter-wave interval (Fig. 3B), mean duration (Fig. 3D), mean amplitude (Fig. 3F), or the spatial correlation of spontaneous Ca^2+^ transients (Fig. 3H). Although significant differences were obtained in the cumulative probability curves for the inter-wave interval, duration, or amplitude (Fig. 3C, E, and G) when comparing to the control or A_2A_R-WT. However, these single-cell effects were diminished by taking the averages across a number of cells and retinas (Fig. 3B, D, and F). Hence, SAC-specific expression of the A_2A_R-ΔC may have relatively minor effects on spontaneous Ca^2+^ transients. These results suggest that the full-length A_2A_R is required for the A_2A_R's up-regulating effects on wave frequency.

### SAC-specific expression of A_2A_R-WT, but not A_2A_R-ΔC, increases the frequency of wave-associated postsynaptic currents or depolarizations in RGCs

**Figure 4:**
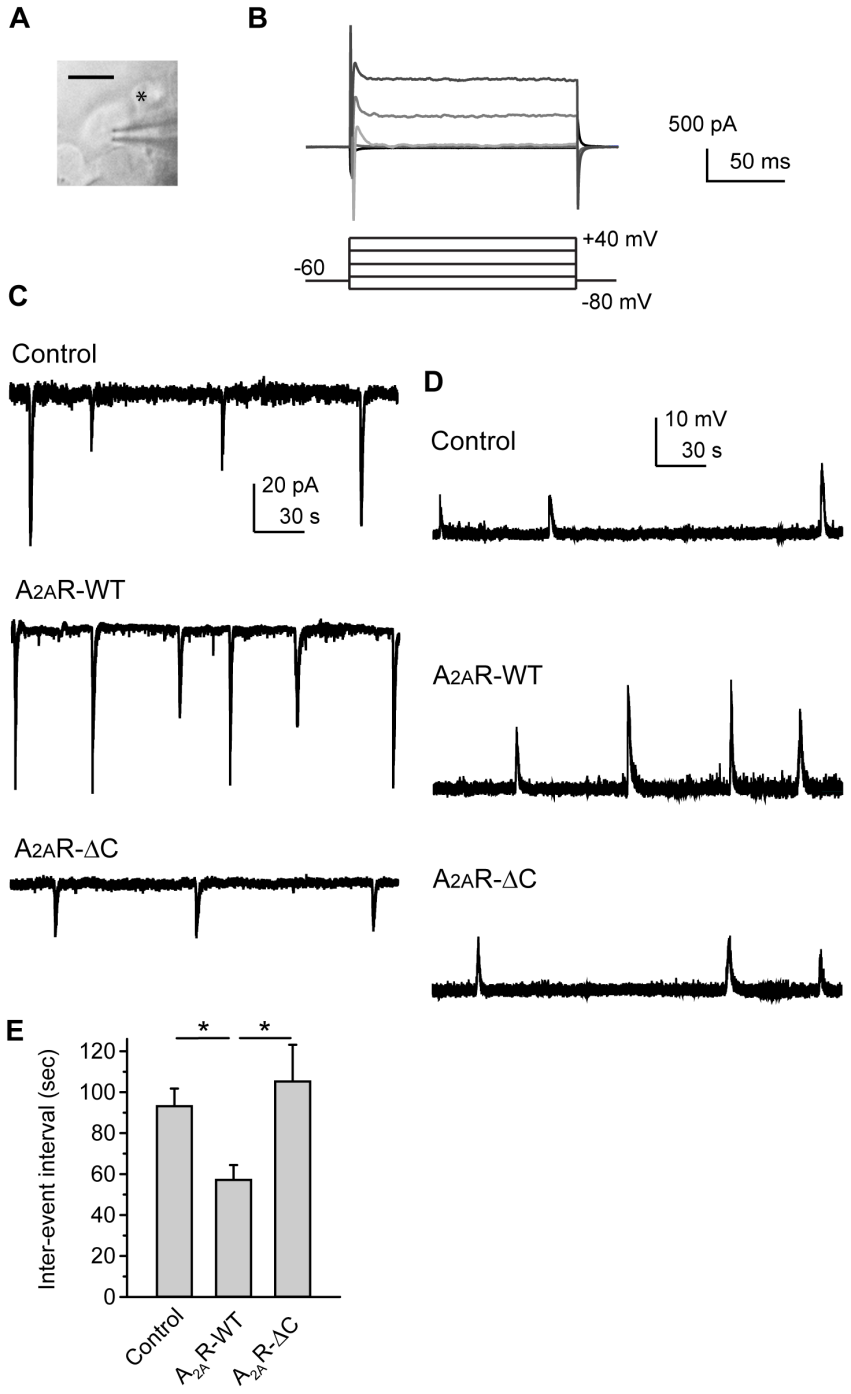
SAC-specific expression of A_2A_R-WT, but not A_2A_R-ΔC, increases the frequency of wave-associated postsynaptic currents or depolarizations. A. The whole-cell patch-clamp recordings with a pipette on a RGC nearby a SAC transfected with pmGluR2-IRES2EGFP. The transfected SAC demonstrated green fluorescence as indicated by the asterisk (*). Scale bar, 10 µm. B. Whole-cell voltage-clamp recordings were used to identify the RGCs, which display the large and quickly-activated Na^+^ currents upon depolarizing voltage pulses [14]. The whole-cell currents from a RGC were induced by stepwise voltage pulses, ranging from −80 to +40 mV with a step size of 30 mV. C. The wave-associated postsynaptic currents (PSCs) were recorded on the RGCs by whole-cell voltage-clamp recordings at the holding potential of −60 mV. The RGCs recorded here were from the retinas previously transfected with Control, A_2A_R-WT, or A_2A_R-ΔC for SAC-specific expression. The PSC's charateristics from different groups were compared in Fig. 5F–I. D. The wave-associated spontaneous depolarizations were recorded on the RGCs from the transfected retinas, using whole-cell current-clamp recordings with no current injected. The depolarization levels from different groups were compared in Fig. 5D–E. E. The inter-event intervals between the wave-associated PSCs (C) or between the spontaneous depolarizations (D) were acquired from the recordings on RGCs out of different transfected groups. Data were obtained from 11–12 recordings on RGCs, 6 transfected retinas, and 6 pups. * *p*<0.05; Kruskal-Wallis method followed by a *post*-*hoc* Dunn test.

To determine how presynaptic A_2A_R affects postsynaptic RGCs, we performed whole-cell patch-clamp recordings on a RGC nearby the transfected SAC (Fig. 4A). The RGCs can be recognized by their relatively large size (10–20 µm) and unique membrane properties [7], i.e., the large Na^+^ currents quickly activated by depolarizing voltage pulses (Fig. 4B). To detect the wave frequency in RGCs, whole-cell voltage-clamp recordings from RGCs revealed wave-associated compound postsynaptic currents (PSCs) (Fig. 4C), reflecting the periodic inputs received by postsynaptic RGCs. We found that presynaptic A_2A_R-WT significantly increased the frequency of wave-associated PSCs in the RGCs (Fig. 4C). Whole-cell current-clamp recordings also revealed that RGCs exhibited wave-associated spontaneous depolarizations more frequently compared to the control (Fig. 4D). Taken together, the inter-event interval of wave-associated PSCs or spontaneous depolarizations in RGCs was significantly decreased by presynaptic A_2A_R-WT (Fig. 4E), suggesting that presynaptic A_2A_R may up-regulate wave frequency in postsynaptic RGCs. By contrast, SAC-specific expression of A_2A_R-ΔC did not change the inter-event interval of wave-associated PSCs or spontaneous depolarizations in RGCs compared to the control (Fig. 4C, D, and E), suggesting that the full-length A_2A_R in SACs is required for up-regulation of wave frequency in RGCs.

### SAC-specific expression of A_2A_R-WT or A_2A_R-ΔC did not alter the membrane properties of postsynaptic RGCs

**Figure 5:**
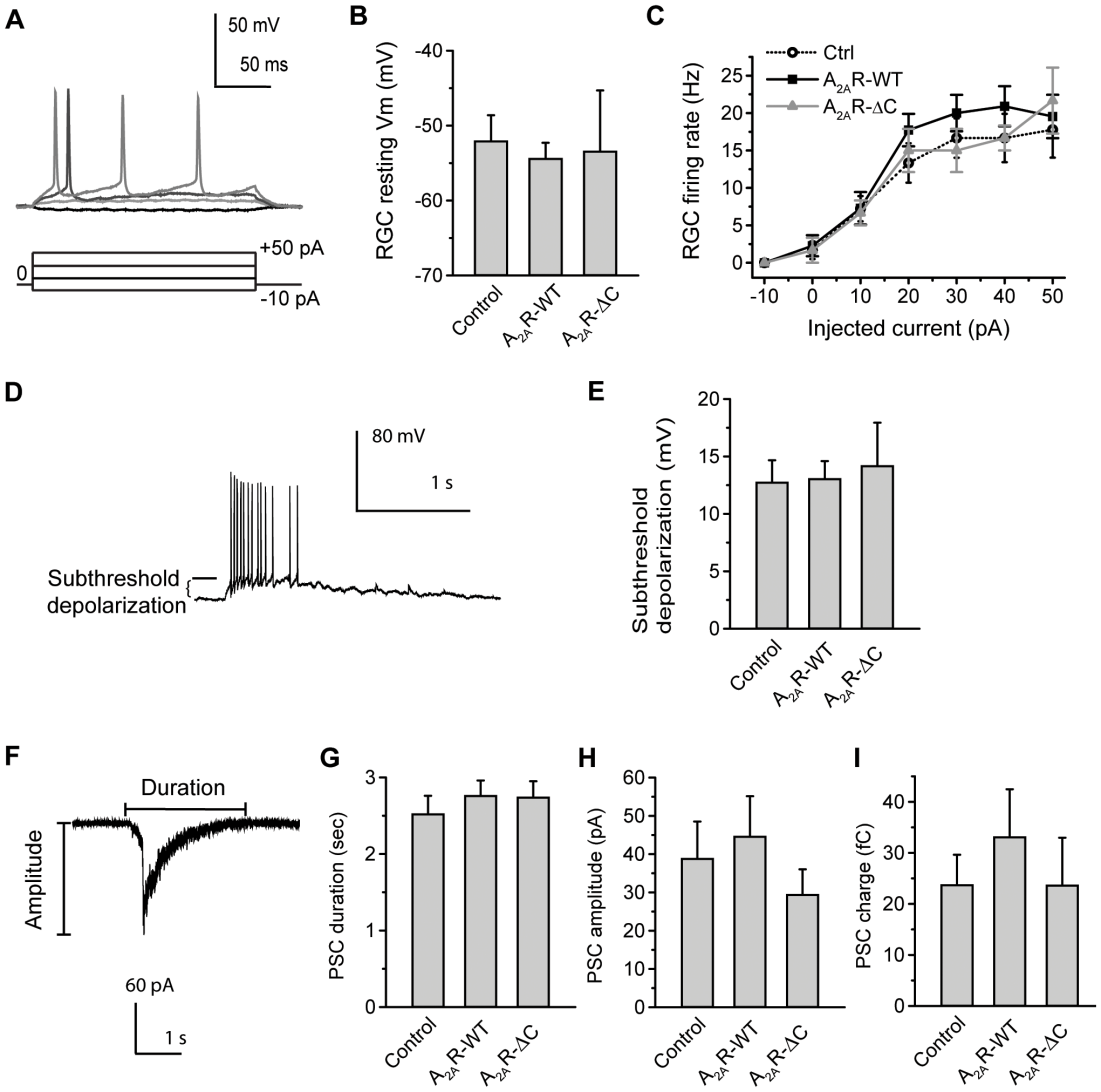
Expression of A_2A_R-WT or A_2A_R-ΔC in presynaptic SACs does not alter the membrane properties of postsynaptic RGCs. A. Representative whole-cell potentials from a RGC induced by 250 msec-current pulses, ranging from −10 to +50 pA with a step size of 20 pA. Note that the action potentials were induced when membrane potentials reached the threshold. B. The resting membrane potentials in RGCs from the retinas transfected by Control, A_2A_R-WT, or A_2A_R-ΔC for SAC-specific expression. Data were obtained from 6–20 RGCs, 6 transfected retinas, and 6 pups. *p* = 0.87; One-way ANOVA with a *post*-*hoc* Student-Newman-Keuls test. C. The firing rate of action potentials in a RGC after SAC-specific expression. Action potentials were induced by injecting various sizes of currents. Data were obtained from 3–11 RGCs, 5–6 transfected retinas, and 5–6 pups. *p* = 0.41–0.53; One-way ANOVA with a *post*-*hoc* Student-Newman-Keuls test. D. Representative wave-associated depolarizations in a RGC revealed by whole-cell current-clamp recordings. The level of subthreshold depolarization was as indicated. E. Summary of subthreshold depolarization in the RGCs after SAC-specific expression. Data were obtained from 14 recordings on RGCs, 5–6 transfected retinas, and 5–6 pups. *p* = 0.91; One-way ANOVA with a *post*-*hoc* Student-Newman-Keuls test. F. A wave-associated PSC in a RGC revealed by whole-cell voltage-clamp recordings. The duration and amplitude of a PSC were as indicated. G. Summary of PSC duration in the RGCs from the transfected retinas. *p* = 0.69; One-way ANOVA with a *post*-*hoc* Student-Newman-Keuls test. H. Summary of PSC amplitude in the RGCs from the transfected retinas. *p* = 0.50; One-way ANOVA with a *post*-*hoc* Student-Newman-Keuls test. I. Summary of PSC charge in the RGCs from the transfected retinas. *p* = 0.73; Kruskal-Wallis method with a *post*-*hoc* Dunn test. For G–I, data were obtained from 8 recordings on RGCs, 5–6 transfected retinas, and 5–6 pups.

Presynaptic A_2A_R up-regulates wave frequency in postsynaptic RGCs. Since the stage-II waves are initiated by SACs [16], [17], [18], [19], presynaptic A_2A_R may not affect the intrinsic excitability of RGCs. To test this hypothesis, we tested the changes in the RGC's excitability after SAC-specific expression. The stepwise current pulses were delivered via a patch pipette to depolarize the RGCs and fire action potentials (Fig. 5A). The resting membrane potentials and firing rate of the RGCs were measured accordingly. We found that presynaptic expression of A_2A_R-WT or A_2A_R-ΔC did not change the resting membrane potential (Fig. 5B) or firing rate (Fig. 5C) of the postsynaptic RGCs, suggesting that presynaptic expression of A_2A_R-WT or A_2A_R-ΔC may not affect the excitability of postsynaptic RGCs. Similarly, there were essentially no membrane potential changes in RGCs upon bath application of an A_2A_R selective agonist (5 µM CGS 21680: ΔVm = −0.3±0.2 mV compared to control; the average RGC's resting membrane potential was −53.2±1.4 mV in the control; N = 4 RGCs) or antagonist (10 µM ZM 241385: ΔVm = −0.1±0.8 mV compared to the control; the average RGC's resting membrane potential was −53.6±1.1 mV in the control; N = 6 RGCs), suggesting that the RGC's membrane properties may not be changed by bath applying A_2A_R drugs. Since bath applying A_2A_R drugs can globally influence the A_2A_R in RGCs but failed to alter RGC's membrane properties, these results were consistent with previous findings that the A_2A_R in RGCs may not play a significant role in setting the periodic rhythms of retinal waves [17], [18].

To further determine whether presynaptic A_2A_R affects the RGC's membrane properties during retinal waves, we measured the levels of subthreshold depolarization during a single event of wave-associated spontaneous depolarization (Fig. 5D). The levels of subthreshold depolarization were not altered by presynaptic A_2A_R-WT or A_2A_R-ΔC (Fig. 5E), suggesting that presynaptic expression of A_2A_R-WT or A_2A_R-ΔC may not affect the RGC's membrane properties during retinal waves.

A_2A_R up-regulates wave frequency in postsynaptic RGCs without altering the RGC's membrane properties, suggesting that the RGC's responsiveness to input signals may not be affected. To examine whether presynaptic A_2A_R alters the amount of input that RGCs receive during waves, we detected the size of wave-associated PSCs. Fig. 5F shows a single wave-associated PSC. The duration (Fig. 5F and G) and peak amplitude (Fig. 5F and H) can be measured from individual PSCs. By integrating the current changes over time, the charge of individual PSCs was acquired (Fig. 5I). We found that presynaptic A_2A_R-WT or A_2A_R-ΔC did not change the PSC duration, amplitude, or charge compared to the control (Fig. 5G–I), suggesting that the amount of input that RGCs receive during waves may not be altered by presynaptic A_2A_R-WT or A_2A_R-ΔC.

### A_2A_R up-regulation of wave frequency may be mediated via the Gs-AC-cAMP pathway

The activation of A_2A_R is thought to stimulate Gs protein and its effector adenylyl cyclase (AC), thereby elevating the intracellular cAMP levels [22]. However, previous studies also suggested that A_2A_R activation may be coupled to various signaling pathways, such as mitogen-activated protein kinase [34], [35], [36], the protein kinase C (PKC) pathway [23], [24], [37], [38], and the interaction with other types of GPCRs, ionotropic receptors, receptor kinases, and adenosine transporters [39]. To determine whether presynaptic A_2A_R up-regulates the wave frequency through the Gs-AC-cAMP pathway, the PKA inhibitor (H89) was bath-applied to the retinas expressing the control vector or A_2A_R-WT in SACs (Figure S2). The inter-wave interval was significantly increased by the PKA inhibitor in the retinas expressing the control vector (Figure S2-A and B). Similarly, a significant increase in the inter-wave interval by H89 treatment was also observed in the retinas expressing A_2A_R-WT in SACs (Figure S2-C and D). Before H89 treatment, the inter-wave interval was significantly decreased in the A_2A_R-WT compared to the control retinas (*p*<0.05; two-tailed Student's unpaired *t*-test). However, the PKA inhibitor can essentially increase the inter-wave interval to the similar levels in both A_2A_R-WT and control (*p* = 0.08; two-tailed Student's unpaired *t*-test). Hence, it suggests that the Gs-cAMP-PKA signaling may be involved in the A_2A_R up-regulation of wave frequency in the A_2A_R-WT over-expressing retinas.

Consistent with the involvement of Gs-AC-cAMP pathway in mediating A_2A_R up-regulation of wave frequency, bath application of selective A_2A_R agonist (CGS 21680) increased both wave frequency and PKA activity (Text S1 and Figure S3). Interestingly, bath application of A_2A_R antagonist (ZM 241385) also increased both wave frequency and PKA activity. These results imply that the ZM 241385 may either act through some other undefined mechanism, or it may not be a pure A_2A_R antagonist in this system. Together, these results suggest that A_2A_R up-regulation of wave frequency may be mediated mainly via the Gs-AC-cAMP pathway.

## Discussion

In this study, we showed that A_2A_R is expressed in the IPL and the GCL of the rat retinas exhibiting stage-II waves. Knockdown of A_2A_R in the postnatal rat retinas decreases the frequency of spontaneous Ca^2+^ transients, suggesting that endogenous A_2A_R up-regulates the frequency of stage-II waves. By utilizing a molecular perturbation method targeted to presynaptic SACs, we tested the effects of presynaptic A_2A_R on spontaneous Ca^2+^ transients, and postsynaptic currents or depolarizations associated with retinal waves. Our results show that presynaptic A_2A_R up-regulates the frequency of stage-II waves in the RGC layer. In contrast, wave frequency is not altered by expressing the C-terminal truncated A_2A_R mutant in SACs, suggesting that the full-length A_2A_R is required for up-regulation of wave frequency. Further, whole-cell current-clamp recordings indicated that presynaptic A_2A_R does not affect the membrane properties of postsynaptic RGCs. Therefore, our results suggest that, during neural circuit refinement, A_2A_R in presynaptic SACs up-regulates the frequency of stage-II waves.

### A_2A_R serves as a positive regulator of retinal wave periodicity: Comparisons between pharmacological experiments and molecular perturbations

Although the importance of adenosine signaling in retinal waves has been recognized for more than ten years, all conclusions have been deduced from pharmacological experiments [3], [14], [20], [21]. The weaknesses of pharmacological experiments limit our understanding of how adenosine signaling regulates retinal waves. For example, bath-applying adenosine reagents does not distinguish their effects on retinal waves through pre- or post-synaptic cells. Moreover, certain adenosine reagents may have unexpected side effects, leading to an incorrect interpretation and conclusion, such as aminophylline acting as a tonic GABA_A_R agonist to regulate retinal waves [3], [14], [21]. We also observed that both A_2A_R selective agonist (CGS 21680) and antagonist (ZM 241385) increased wave frequency and PKA activity (Text S1 and Figure S3). In our study based on molecular perturbation (knockdown or SAC-specific expression), we found that A_2A_R up-regulates the frequency of stage-II waves, similar to the results by either an A_2A_R selective agonist (CGS 21680) (Figure S3) or the A_2_R general agonist NECA [20]. Together with the previous pharmacological results [3], [14], [20], [21], our present study suggests that the activation of A_2A_R may up-regulate the frequency of retinal waves. Hence, endogenous adenosine binding to A_2A_R may serve as a positive regulator of wave periodicity.

### Presynaptic SACs mediate A_2A_R up-regulation of wave frequency via the cAMP-dependent pathway

Previous studies have implied that adenosine may act through presynaptic SACs to increase the frequency of retinal waves [14], [20], but direct evidence is currently missing. In this study, we employed the mGluR2 promoter to target A_2A_R expression in SACs and detect the subsequent changes in Ca^2+^ transient frequency. Knockdown of A_2A_R decreases wave frequency (Fig. 2). SAC-specific expression of the A_2A_R-WT increases wave frequency (Fig. 3), but this effect is not observed by non-SAC-specific expression of the A_2A_R-WT (Table S1). Thus, it suggests that A_2A_R up-regulation of wave frequency may act via presynaptic SACs.

Additional evidence supporting the role of presynaptic A_2A_R in up-regulating wave frequency is provided by the results of patch-clamp recordings on RGCs. We found that presynaptic A_2A_R did not affect RGC's excitability, membrane potentials, or PSC charge (Fig. 5), suggesting that postsynaptic RGCs may not undergo the changes in membrane properties for A_2A_R up-regulation of wave frequency. Our pharmacological results from A_2A_R agonist or antagonist also support this conclusion. Taken together, these data are consistent with previous findings that RGCs are the output neurons participating in, but not initiating, stage-II waves [2], [16], [17], [18], [19].

In the present study, we found that activation of the Gs-AC-cAMP pathway is important for A_2A_R up-regulation of wave frequency (Figure S2). One previous study by Zheng et al. has clearly shown that bursting in SACs depends upon the cAMP-sensitive potassium current [18]. Hence, the most obvious explanation of adenosine effects described here or elsewhere [3], [14], [20], [21] is that the adenosine modulates the bursting frequency in SACs through the Gs-AC-cAMP pathway. Consistent with this explanation, A_2A_R has been shown to modulate neurotransmitter release in the nervous system [39]. Developing SACs co-release ACh and GABA during stage-II waves [17]. According to our results using whole-cell voltage-clamp recordings (Fig. 4C and 5F–I), perturbations of presynaptic A_2A_R altered the frequency but not the amount of inputs that RGCs received (mainly cholinergic input [17], [18]). Together with the results demonstrating no significant changes in the RGC's membrane properties (Fig. 5), we suggest that A_2A_R up-regulation of wave frequency most likely takes place on presynaptic release. Therefore, the activation of A_2A_R in the developing SACs probably leads to an increase in the bursting frequency and then neurotransmitter release, thus enhancing wave frequency.

### A_2A_R in postsynaptic RGCs

Although A_2A_R is also expressed in RGCs (Fig. 1), the function mediated through RGCs remains to be identified. Here, we cannot exclude the possibility that RGCs may affect other spatiotemporal properties of stage-II waves, such as the propagation speed. A previous study showed that globally increasing the intracellular cAMP level or PKA activity can increase the size, speed, and frequency of stage-II waves [20]. However, our results indicated that the A_2A_R in SACs did not affect the spatial correlation of stage-II waves (Fig. 3), suggesting that A_2A_R in SACs may not play a critical role in the propagation of retinal waves. By contrast, bath applying an A_2A_R selective agonist enhanced the PKA activity in developing retinal neurons (most of which may be RGCs) (Figure S3-C and E). These results imply that activation of A_2A_R in RGCs may amplify the Gs-AC-cAMP signaling *in situ*, possibly leading to the increased size or speed of retinal waves. Whether the A_2A_R in RGCs is critical for wave propagation requires further investigation.

### Other signaling pathways underlying activation of A_2A_R

In the present study, we found that the C-terminal truncated A_2A_R mutant (A_2A_R-ΔC) cannot increase the wave frequency (Fig. 3–5), suggesting that the C-terminus of A_2A_R may play a role in up-regulating the wave frequency. The long intracellular C-terminus is only present in A_2A_R but not other adenosine receptor subtypes [24]. Previous pharmacology experiments showed that wave frequency is not altered by blocking other adenosine receptor subtypes [14], consistent with the role of the C-terminus in mediating the A_2A_R up-regulation of retinal waves. The C-terminus of A_2A_R has been shown to regulate both cAMP-dependent and -independent signaling pathways [25], [26], [33]. The C-terminal segment deleted in A_2A_R-ΔC can interact with the Gas-2 like 2, which facilitates the recruitment of the trimeric G protein complex and couples the Gαs-mediated cAMP signaling [25]. This C-terminal segment also interacts with the translin-associated protein X (TRAX), a DNA-binding protein that modulates axonal regeneration by regulating gene expression [26], [33], [40]. We found that the Gs-AC-cAMP pathway may be important for A_2A_R up-regulation of wave frequency (Figure S2). Whether the cAMP-independent mechanisms also participate in the A_2A_R up-regulation of wave frequency requires further investigation.

### Future directions

Our study first demonstrates that A_2A_R up-regulates the frequency of stage-II waves via “presynaptic” SACs, thereby providing a potential target for the manipulation of retinal waves. Stage-II waves are present from P0 until P9 in rats [13], [14], and we also found that A_2A_R is expressed in the IPL and GCL until P9, suggesting that A_2A_R may up-regulate stage-II waves across the entire period. Previous studies have found that stage-II waves propagate through the developing retina, inducing similar burst patterns in the thalamus and visual cortex to form sensory loops [2], [12]. Hence, disturbing A_2A_R signaling in presynaptic SACs may alter the periodic rhythms of stage-II waves, possibly resulting in defective retinogeniculate and retinocollicular projections [5], [6], [9], [10], [11], [30]. How the A_2A_R in SACs may regulate retinogeniculate and retinocollicular projections requires further investigation. Thus, the results pertaining to the role of A_2A_R in regulating retinal waves would provide new insights to develop therapeutic methods for related diseases [41], [42].

## Materials and Methods

### Molecular Biology

To knockdown endogenous A_2A_R in rat retinal explants, the siRNA (TTA CAT GGT TTA CTA CAA C) designed for rat A_2A_R was carried by the vector pSuper-hrGFP (OligoEngine #VEC-PBS-0006). Transfected cells were identified by green fluorescence from the expression of humanized recombinant green fluorescent protein (hrGFP).

The vector pmGluR2-IRES2EGFP was used for SAC-specific gene expression in the retinal explant culture [28]. This vector contains a mGluR2 promoter [14], [31], [32] and an internal ribosome entry site (IRES), allowing proteins of interest and enhanced green fluorescent protein (EGFP) to be translated separately from the same strand of coding mRNA. Thus, transfected SACs can be identified by the EGFP green fluorescence [28]. In this study, the cDNAs encoding rat wild-type A_2A_R [43] or the C-terminal truncated A_2A_R (A_2A_R_1–322_) [25], [26], [33] were subcloned into pmGluR2-IRES2EGFP with *Bgl* II and *Sal* I sites. Successful constructs were confirmed by sequencing.

### Ethics Statement and Animals

This study was carried out in strict accordance with the recommendations in the Guide for the Care and Use of Laboratory Animals of the National Institutes of Health. The protocol for all animal experiments was approved by the Institutional Animal Care and Use Committee of National Taiwan University (Permit Number: 96–49 and 97–27). Postnatal (P1–P2) Sprague-Dawley rats (BioLASCO, Taiwan) were used in this study. All rat pups were housed with their own mothers in individually ventilated cages with well-controlled conditions (12:12 light/dark cycle with lights on at 7 AM; 20±1°C) and *ad libitum* access to food and water. Rat pups (P1–P2) were deeply anesthetized by hypothermia before decapitation, with all efforts to minimize suffering.

### Retinal Explant Culture and Transient Transfection

All procedures for retinal explant culture and transient transfection followed our previously described methods [28]. Briefly, the whole-mount retina from postnatal rat pups was isolated in dissection buffer [1× HBSS (GIBCO), 10 mM HEPES, and 0.35 g/L NaHCO_3_, pH 7.35] and attached onto nitrocellulose membranes (Millipore) with the RGC layer facing up. DNA plasmids (200 ng/µL in dissection buffer) carrying the siRNA or encoding the proteins of interest were transfected into retinal explants by electroporation using our homemade horizontal electrodes (27 V, 4 mm, 50 ms of pulse duration, 2 pulses at 1 sec-interval; BTX ECM830 electroporator) [28]. Retinal explants were subsequently cultured for 60–96 hr at 35°C in a 5% CO_2_ humidified incubator, and daily supplied with fresh Retinal Serum-Free Culture Medium (SFCM-A) containing Neurobasal-A (GIBCO #10888), 0.6% Glucose, 2 mM L-Glutamine (Sigma #G6392), 1× B-27 (GIBCO #17504-044), 10 mM HEPES, 1 mM Sodium Pyruvate (GIBCO #11360-070), 2.5 µg/mL Insulin (Sigma #I1882), 100 µg/mL Penicillin/100 units/mL Streptomycin (GIBCO #15140-122), and 6 µM Forskolin [13].

### Immunofluorescence

For retinal cross-section staining, the deeply anesthetized pups were perfused with 4% paraformaldehyde (PFA). The eyeballs were isolated and post-fixed by 4% PFA at 4°C overnight, followed by cryoprotection in 30% sucrose for 2 d and preservation in optical cutting temperature (O.C.T.) gel (Sakura Finetech #4583). Retinal cross-sections (16 µm) were prepared with a cryostat (Leica CM1850), placed on poly-lysine-coated slides, and blocked at RT for 1 hr in 3% donkey-serum blocking solution (DBS), consisting of 3% Donkey serum (Jackson Lab #017000121), 0.5% Triton X-100, and 0.1% sodium azide in phosphate buffered saline (PBS) [28]. Retinal cross-sections or dissociated SACs [13] were first incubated with primary antibodies in 1% DBS [mouse monoclonal anti-A_2A_R (1∶800; Millipore #05-717) and goat polyclonal anti-ChAT (1∶200; Millipore #AB144P)] at 4°C overnight, washed with PBS, further incubated with secondary antibodies in 1% DBS [donkey-anti-mouse IgG conjugated with Alexa Fluor 488 (1∶800 Invitrogen #A21202) and donkey-anti-goat IgG conjugated with Alexa Fluor 568 (1∶800; Invitrogen #A11057)] at RT for 2 hr, and washed with PBS again. The samples were finally stained with DAPI at RT for 10 min.

For whole-mount retina staining, the retinal explants were placed on poly-lysine-coated slides, fixed with 4% PFA at RT for 30 min, and washed with PBS for 1 hr. Retinal explants were blocked in 3% DBS at RT for 1 hr, incubated with the primary antibody in 1% DBS [mouse monoclonal anti-A_2A_R (1∶800; Millipore); or together with goat polyclonal anti-ChAT (1∶200; Millipore)] at RT for 2 days, washed with PBS, further incubated with the secondary antibody in 1% DBS (donkey-anti-mouse IgG conjugated with Alexa Fluor 488; or together with donkey-anti-goat IgG conjugated with Alexa Fluor 568) at RT overnight, and washed with PBS again.

For immunostaining of dissociated retinal cells, the cells were dissociated from retinas, plated on the coverslips, washed with PBS at RT, fixed with 4% PFA at RT for 15 min, and washed with PBS at RT for 20 min. After fixation, the dissociated cells was incubated with 0.1% Triton X-100 at RT for 10 min and washed with PBS at RT for 30 min. The dissociated cells were blocked in 3% DBS with 0.1% Triton X-100 at RT for 1 hr, incubated with the primary antibodies in 3% DBS [goat polyclonal anti-ChAT (1∶50; Millipore) and mouse monoclonal anti-A_2A_R (1∶400; Millipore)] at 4°C overnight, washed with PBS, further incubated with the secondary antibodies in 3% DBS [donkey-anti-goat IgG conjugated with Alexa Flour 647 (1∶500; Invitrogen #A11057) and donkey-anti-mouse conjugated with Dylight 549 (1∶500; Jackson ImmunoResearch #715-505-151)] at RT for 2 hr, and washed with PBS at RT for 1 hr.

The anti-fade reagent Fluoromount G (Electron Microscopy Sciences #17984-25) was added to the samples on the slides before sealing with coverslips. Fluorescent images were acquired by confocal microscopy (Leica TCS SP5 spectral) and quantitatively analyzed using MetaMorph software (Version 7.5, Molecular Devices). The EGFP fluorescence was used to detect transfected SACs.

### Western Blot Analysis

To assess the siRNA knockdown efficiency, PC12 cells were transfected with an empty vector pSuper-hrGFP, or pSuper-hrGFP with siRNA against A_2A_R using Lipofectamine 2000 (Invitrogen) [33]. At 36 hr post transfection, cells were sorted by the expression of hrGFP and dissolved in ice-cold RIPA buffer (150 mM NaCl, 10 mM sodium phosphate, 1% Triton X-100, 0.5% sodium deoxycholate, pH 7.2, supplemented with protease inhibitor cocktail). The protein concentration was determined by a Bradford assay (Bio-Rad #500-0006). Protein in the cellular lysates was electrophoresed through standard 10% Laemmli SDS polyacrylamide gel, transferred to polyvinylidene difluoride membrane (Millipore), blocked with 5% skim milk in TBST (0.2 M Tris-base, 1.37 M NaCl, and 0.05% Tween 20), and then incubated with primary antibodies [mouse monoclonal anti-A_2A_R (Millipore) and mouse anti-α-tubulin (Sigma #T5168)] at 4°C overnight. Membranes were washed three times with TBST and then incubated with the horseradish peroxidase-conjugated secondary antibodies (1∶5000; GE Health-care) at RT for 30 min [33]. Membranes were washed three times with TBST, and the immunoreactive bands were visualized using a light-emitting nonradioactive method (ECL, Amersham, Bucks, UK).

### Live Ca^2+^ Imaging and Data Analysis

Live Ca^2+^ imaging and data analysis were performed in transfected retinal explants as described previously [28]. Briefly, the cultured explants were transferred to forskolin-free SFCM-A and loaded with the Ca^2+^ indicator fura-2-AM (Molecular Probes #F1221) by a standard protocol [28]. During imaging, the explants were continuously perfused with artificial cerebrospinal fluid [ACSF, 119 NaCl, 26.2 NaHCO_3_, 2.5 KCl, 1.0 K_2_HPO_4_, 1.3 MgCl_2_, 2.5 CaCl_2_, and 11 D-glucose (in mM)] bubbled with 95% O_2_/5% CO_2_ warmed to 30°C. Live Ca^2+^ imaging was performed on an upright fluorescent microscope (Olympus BX51WI) with a 20× water immersion objective. The transfected cells were identified by EGFP fluorescence (Ex 470/Em 525, Chroma #D41017). The fura-2 fluorescence was excited at 380 nm (Chroma #D380xv2) via a xenon arc lamp (DG-4, Sutter Instrument) with a dichoic mirror (455DCLP, Chroma); it was captured at 510 nm (Chroma #D510/40 m) by a CCD camera (CoolSNAP HQ2, Photometrics) at 1 s-intervals for 10 min, with 100–150 ms exposure times.

Digitized imaging data for individual cells were acquired from the fluorescence changes across all time frames, previously background subtracted for each frame by MetaMorph. An Igor (WaveMetrics) procedure written in this laboratory was used to correct baseline photobleaching and unbiasedly analyze the characteristics of spontaneous Ca^2+^ transients, including the duration, amplitude, and inter-wave interval for the measurement of wave frequency [28]. Further data analysis was conducted by Excel and Origin 8 (OriginLab). The mean data for one transfection group were averaged from all Ca^2+^ transients in each cell, across 10 cells out of one imaged region (340×460 µm), then from two imaged regions out of one retina, and finally, from all retinas transfected with the same gene. Distributions of cumulative probability were constructed from single-cell data for the same transfection group. Correlation of spontaneous Ca^2+^ transients between nearby cells was evaluated by the correlation index (C.I.) [29], [30] according to the following equation:
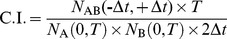

*N*_AB_ is the transient number for which cell B exhibits within a time window ± Δ*t* (3 sec) from cell A, *N*_A_ and *N*_B_ are the total numbers of transients exhibited by cells A and B, respectively, during the total recording time (*T*, 600 sec). The averaged C.I. values were computed from the same distance group and plotted against the intercellular distance according to our described methods [28].

### Electrophysiology

Whole-cell patch-clamp recordings were performed on visualized RGCs (60× water-immersion objective, Olympus) in transfected retinal explants that were continuously superfused with oxygenated ACSF at 30°C, as described in the previous studies [13], [14]. Borosilicate glass pipettes (WPI #PG52151-4) were pulled (Narishige PC-10) to a tip resistance of ∼5.5 MΩ when filled with a pipette solution [98.3 K-gluconate, 1.7 KCl, 0.6 EGTA, 5 MgCl_2_, 40 HEPES, 2 Na_2_-ATP, 0.3 Na-GTP (in mM), pH 7.25 with KOH]. Recordings were made using an Axopatch 200B patch-clamp amplifier with Digidata 1440A interphase (Molecular Devices). Data were acquired and analyzed with the pClamp10 software (Molecular Devices). For whole-cell voltage-clamp recordings, the current responses (filtered at 1 kHz and digitized at 5 kHz) were recorded at a holding potential of −60 mV, or with other protocols indicated in the figure legends. For whole-cell current-clamp recordings, the membrane potential changes (filtered at 5 kHz and digitized at 10 kHz) were monitored with no current injected unless indicated elsewhere. In successful recordings, gigaohm seals were obtained within 30 s, and the ratios of access resistance to input resistance were 5–15%. The mean data for the same transfection group were averaged from all events in each cell and were then averaged across a number of cells transfected with the same gene.

### Statistics

All data were presented as the mean ± S.E.M. Statistical significance was evaluated for two groups by the two-tailed Student's unpaired *t*-test as the parametric method, or the Mann-Whitney method as the nonparametric method. For three groups, statistical significance was evaluated using One-way ANOVA with a *post*-*hoc* Student-Newman-Keuls test as the parametric method, or the Kruskal-Wallis method with a *post*-*hoc* Dunn test as the nonparametric method. The Kolmogorov-Smirnov test was used to evaluate significant differences between the cumulative probabilities of different groups. Asterisks indicated significance in the following manner: *, *p*<0.05; **, *p*<0.01 (InStat 3, GraphPad).

## Supporting Information

Figure S1 **Immunofluorescence staining of A_2A_R after targeting expression to SACs by the mGluR2 promoter.** Immunofluorescence staining of A_2A_R (green) and ChAT (red) in the P2 whole-mount retinas expressing either (A) control vector (pmGluR2-IRES2EGFP), (B) A_2A_R-WT (pmGluR2-IRES2EGFP-wild-type A_2A_R), or (C) A_2A_R-ΔC (pmGluR2-IRES2EGFP-C-terminal-deletion mutant of A_2A_R). D–F. The high magnification of the images in the respective boxes of A–C. For A–F confocal images, the z-section thickness was 0.77 µm. G. Immunofluorescence staining of A_2A_R (green) and ChAT (red) in single SACs dissociated from the retinas expressing either control vector, A_2A_R-WT, or A_2A_R-ΔC. *Right*, the merged images under the bright field. The colocalization of A_2A_R and ChAT immunoreactivities was shown in yellow. Scale bars for A–C, 15 µm. Scale bars for D–F, 5 µm. Scale bars for G, 7.5 µm. H. Quantification of A_2A_R immunoreactivity in the dissociated SACs from different transfected groups (N = 6–23). ** *p*<0.01; One-way ANOVA with a *post*-*hoc* Student-Newman-Keuls test. Note that ChAT immunoreactivity was comparable in all groups. *p* = 0.24; Kruskal-Wallis method with a *post*-*hoc* Dunn test.
(PDF)

Figure S2 **Inhibition of PKA activity reduces the Ca^2+^ transient frequency in the retinas expressing A_2A_R-WT in SACs.** (A) Representative traces of spontaneous Ca^2+^ transients from the retina expressing the control vector (pmGluR2-IRES2EGFP) in the absence or presence of the PKA inhibitor (50 µM H89 for 10 min). (B) The inter-wave interval for correlated Ca^2+^ transients was compared before and after the PKA inhibitor treatment in the same cells from the control group. (C) Representative traces of spontaneous Ca^2+^ transients from the retina expressing the A_2A_R-WT in SACs (pmGluR2-IRES2EGFP-wild-type A_2A_R) in the absence or presence of the PKA inhibitor (50 µM H89 for 10 min). (D) The inter-wave interval was compared before and after the PKA inhibitor treatment in the same cells from the A_2A_R-WT group. Data were obtained from 5–7 transfected retinas. **p<0.01; two-tailed Student's unpaired *t*-test.
(PDF)

Figure S3 **Both wave frequency and PKA activity are increased by the selective A_2A_R agonist or antagonist.** (A) Wave-associated depolarizations in the absence (Control) and presence of A_2A_R agonist (Ai, 5 µM CGS 21680) or antagonist (Aii, 10 µM ZM 241385). (B) The average changes in the inter-event interval of wave-associated depolarizations in the presence or absence of the A_2A_R agonist or antagonist. Data were normalized to the control. Data were obtained from 4–6 acutely isolated P2 rat retinas. * *p*<0.05; Mann-Whitney method for comparing the presence and absence of the A_2A_R agonist; * *p*<0.05; two-tailed Student's unpaired *t*-test for comparing the presence and absence of the A_2A_R antagonist. (C) Ci, a cell expressing the FRET-based PKA activity reporter in the CFP or YFP channel [44], [45], [46], [47], [48], [49], [50], [51], [52]. Scale bar for both channels, 10 µm. Cii and Ciii, changes in the fluorescence intensity (ΔF) (green traces, acquired from the CFP channel; yellow traces, acquired from the YFP channel); changes in the FRET ratios (ΔR) (black traces) [13], [44], [45], [46], [47], [48], [49], [53], [54] upon application of an A_2A_R agonist or antagonist. (D) The FRET ratios before, during and after the application of an A_2A_R agonist (Di) or antagonist (Dii). Black, FRET ratios from individual cells. Red, average FRET ratios from cells. (Di) *p<0.05, repeated measures ANOVA; (Dii) **p<0.01, Friedman test. (E) The average changes in the FRET ratios (ΔR) induced by an A_2A_R agonist or antagonist. Each circle indicates the experiment from one cell. Data were obtained from 5–6 cells, 3 transfected retinas, and 3 pups. *p* = 0.51; two-tailed Student's unpaired *t*-test.
(PDF)

Table S1 **Ca^2+^ transient characteristics following expression of A_2A_R under the control of the CMV promoter.**
(PDF)

Text S1 **Methods: Fluorescence resonance energy transfer (FRET) imaging and Pharmacology.**
(PDF)
