# Correction: Adenosine A_2A_ Receptor Up-Regulates Retinal Wave Frequency via Starburst Amacrine Cells in the Developing Rat Retina

**DOI:** 10.1371/journal.pone.0314497

**Published:** 2024-11-20

**Authors:** Pin-Chien Huang, Yu-Tien Hsiao, Shao-Yen Kao, Ching-Feng Chen, Yu-Chieh Chen, Chung-Wei Chiang, Chien-fei Lee, Juu-Chin Lu, Yijuang Chern, Chih-Tien Wang

In Fig 5, the unit in the Y-axis of Fig 5I should be “pC”, not “fC”. Please see the correct Fig 5 here.

**Fig 5:**
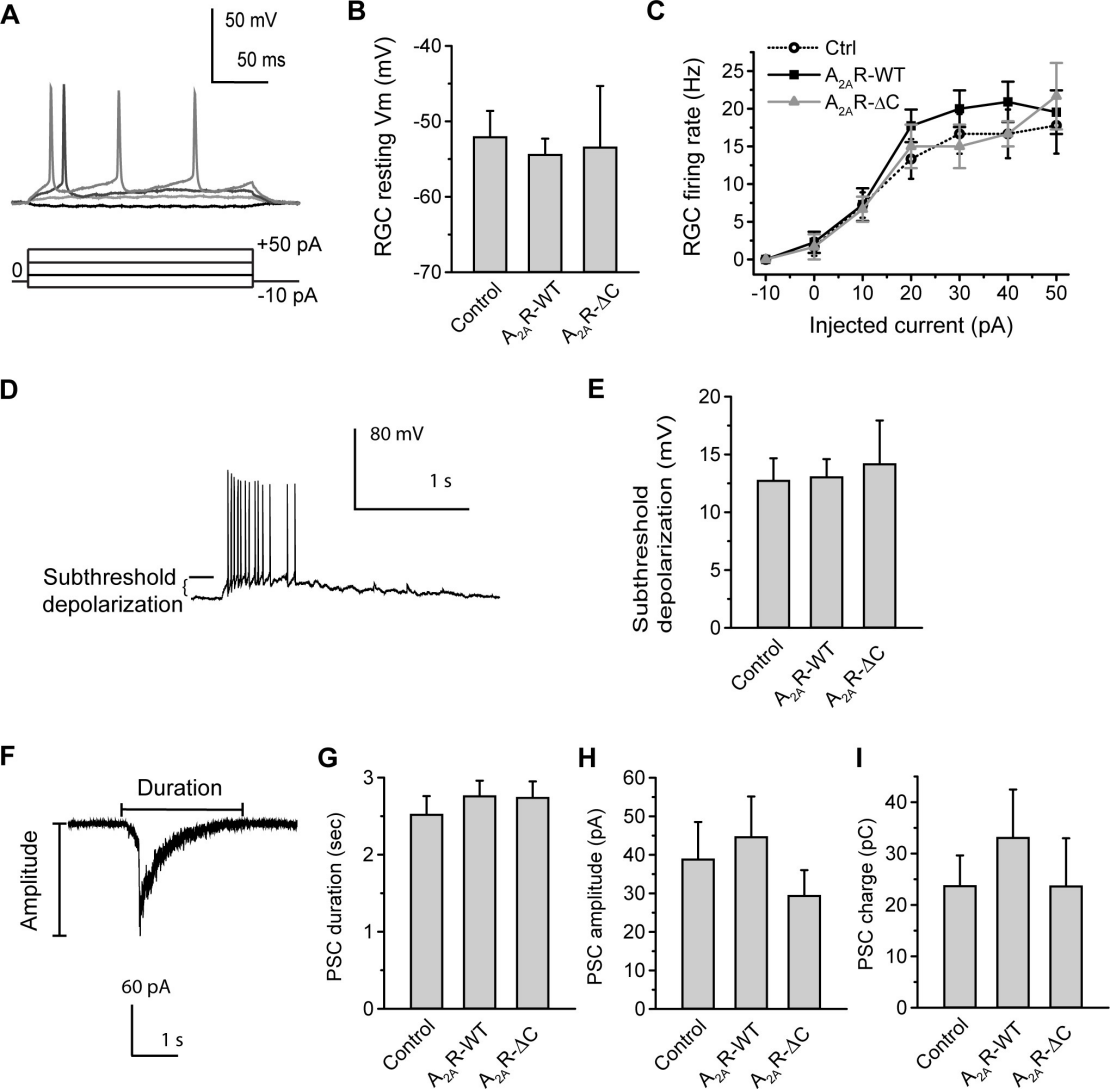
Expression of A2AR-WT or A2AR-ΔC in presynaptic SACs does not alter the membrane properties of postsynaptic RGCs. A. Representative whole-cell potentials from a RGC induced by 250 msec-current pulses, ranging from −10 to +50 pA with a step size of 20 pA. Note that the action potentials were induced when membrane potentials reached the threshold. B. The resting membrane potentials in RGCs from the retinas transfected by Control, A2AR-WT, or A2AR-ΔC for SAC-specific expression. Data were obtained from 6–20 RGCs, 6 transfected retinas, and 6 pups. p = 0.87; One-way ANOVA with a post-hoc Student-Newman-Keuls test. C. The firing rate of action potentials in a RGC after SAC-specific expression. Action potentials were induced by injecting various sizes of currents. Data were obtained from 3–11 RGCs, 5–6 transfected retinas, and 5–6 pups. p = 0.41–0.53; One-way ANOVA with a post-hoc Student-Newman-Keuls test. D. Representative wave-associated depolarizations in a RGC revealed by whole-cell current-clamp recordings. The level of subthreshold depolarization was as indicated. E. Summary of subthreshold depolarization in the RGCs after SAC-specific expression. Data were obtained from 14 recordings on RGCs, 5–6 transfected retinas, and 5–6 pups. p = 0.91; One-way ANOVA with a post-hoc Student-Newman-Keuls test. F. A wave-associated PSC in a RGC revealed by whole-cell voltage-clamp recordings. The duration and amplitude of a PSC were as indicated. G. Summary of PSC duration in the RGCs from the transfected retinas. p = 0.69; One-way ANOVA with a post-hoc Student-Newman-Keuls test. H. Summary of PSC amplitude in the RGCs from the transfected retinas. p = 0.50; One-way ANOVA with a post-hoc Student-Newman-Keuls test. I. Summary of PSC charge in the RGCs from the transfected retinas. p = 0.73; Kruskal-Wallis method with a post-hoc Dunn test. For G–I, data were obtained from 8 recordings on RGCs, 5–6 transfected retinas, and 5–6 pups.
