# Supplementary figures and images for: Adenosine A2A Receptor Up-Regulates Retinal Wave Frequency via Starburst Amacrine Cells in the Developing Rat Retina
Source: PLoS One. 2014 Apr 28;9(4):e95090. doi: 10.1371/journal.pone.0095090 (PMC4002430; doi:10.1371/journal.pone.0095090)

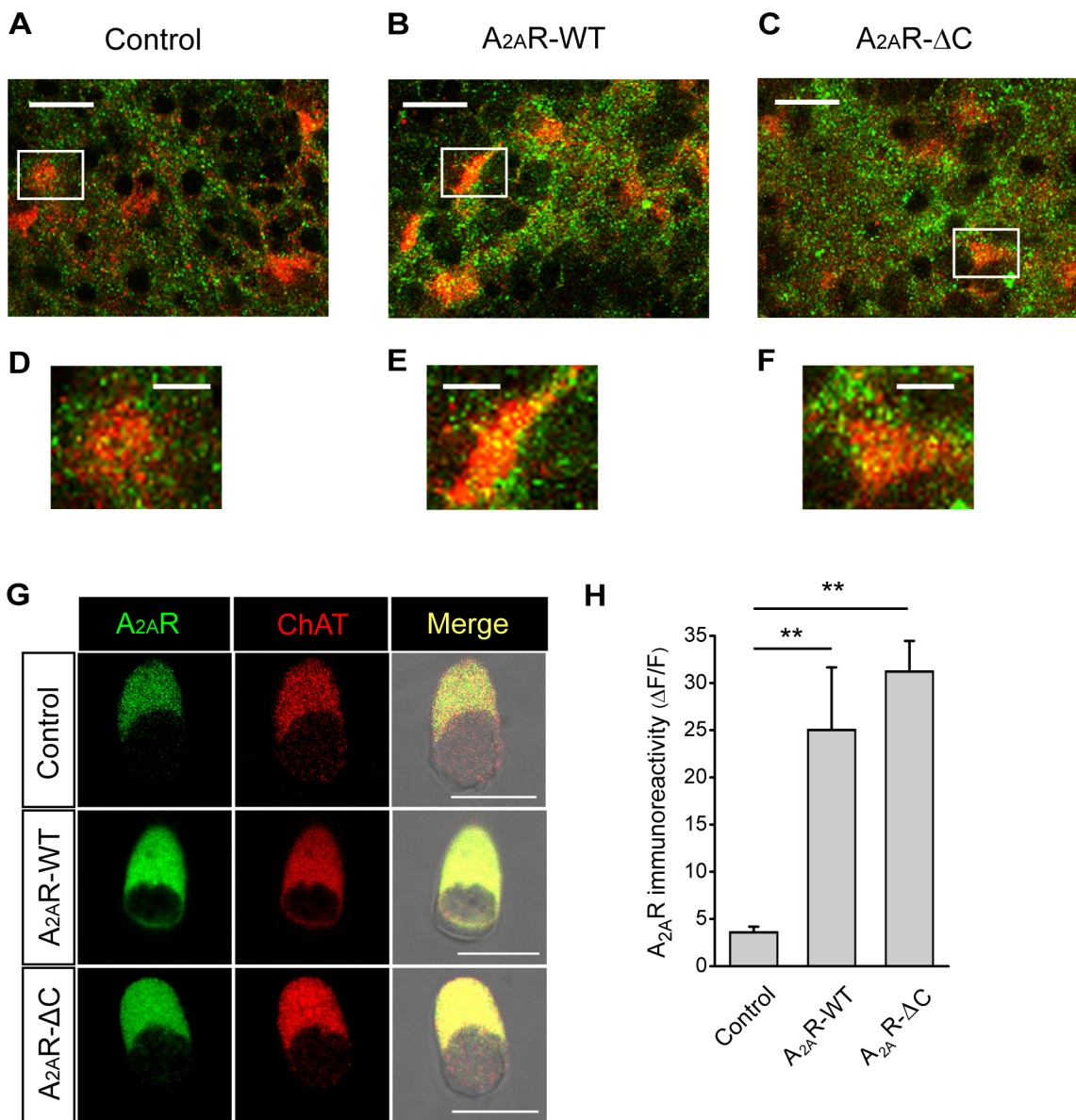

**Figure S1**

Supplement: Figure S1 — Immunofluorescence staining of A2AR after targeting expression to SACs by the mGluR2 promoter. Immunofluorescence staining of A2AR (green) and ChAT (red) in the P2 whole-mount retinas expressing either (A) control vector (pmGluR2-IRES2EGFP), (B) A2AR-WT (pmGluR2-IRES2EGFP-wild-type A2AR), or (C) A2AR-ΔC (pmGluR2-IRES2EGFP-C-terminal-deletion mutant of A2AR). D–F. The high magnification of the images in the respective boxes of A–C. For A–F confocal images, the z-section thickness was 0.77 µm. G. Immunofluorescence staining of A2AR (green) and ChAT (red) in single SACs dissociated from the retinas expressing either control vector, A2AR-WT, or A2AR-ΔC. Right, the merged images under the bright field. The colocalization of A2AR and ChAT immunoreactivities was shown in yellow. Scale bars for A–C, 15 µm. Scale bars for D–F, 5 µm. Scale bars for G, 7.5 µm. H. Quantification of A2AR immunoreactivity in the dissociated SACs from different transfected groups (N = 6–23). ** p<0.01; One-way ANOVA with a post-hoc Student-Newman-Keuls test. Note that ChAT immunoreactivity was comparable in all groups. p = 0.24; Kruskal-Wallis method with a post-hoc Dunn test. (PDF) [file pone.0095090.s001.pdf]

**A**

Control

- H89

+ H89

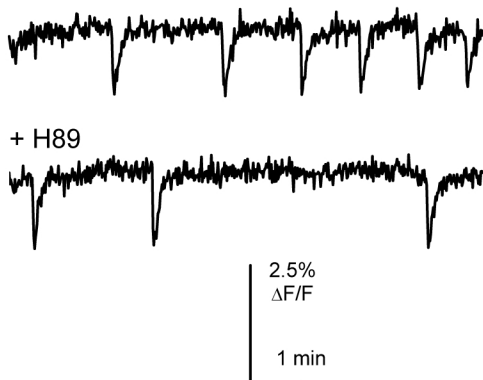**B**

Control

**\*\***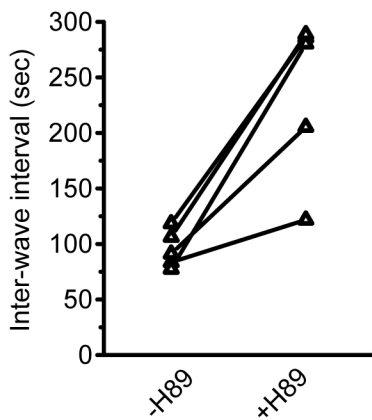**C** $A_{2A}R$ -WT

- H89

+ H89

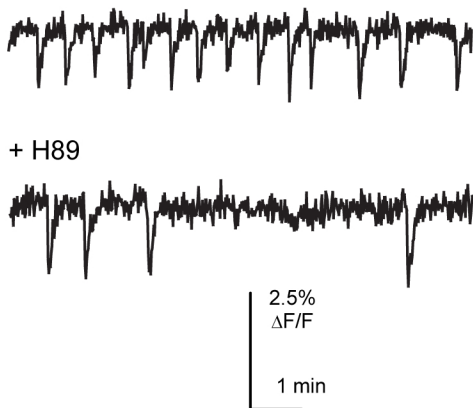**D** $A_{2A}R$ -WT**\*\***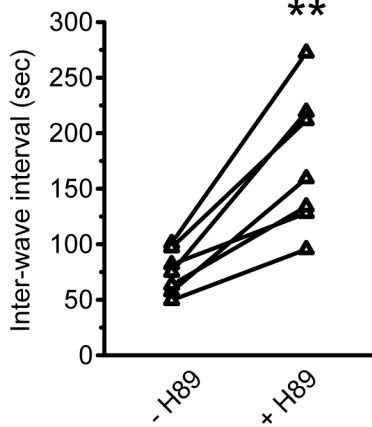**Figure S2**

Supplement: Figure S2 — Inhibition of PKA activity reduces the Ca2+ transient frequency in the retinas expressing A2AR-WT in SACs. (A) Representative traces of spontaneous Ca2+ transients from the retina expressing the control vector (pmGluR2-IRES2EGFP) in the absence or presence of the PKA inhibitor (50 µM H89 for 10 min). (B) The inter-wave interval for correlated Ca2+ transients was compared before and after the PKA inhibitor treatment in the same cells from the control group. (C) Representative traces of spontaneous Ca2+ transients from the retina expressing the A2AR-WT in SACs (pmGluR2-IRES2EGFP-wild-type A2AR) in the absence or presence of the PKA inhibitor (50 µM H89 for 10 min). (D) The inter-wave interval was compared before and after the PKA inhibitor treatment in the same cells from the A2AR-WT group. Data were obtained from 5–7 transfected retinas. **p<0.01; two-tailed Student's unpaired t-test. (PDF) [file pone.0095090.s002.pdf]

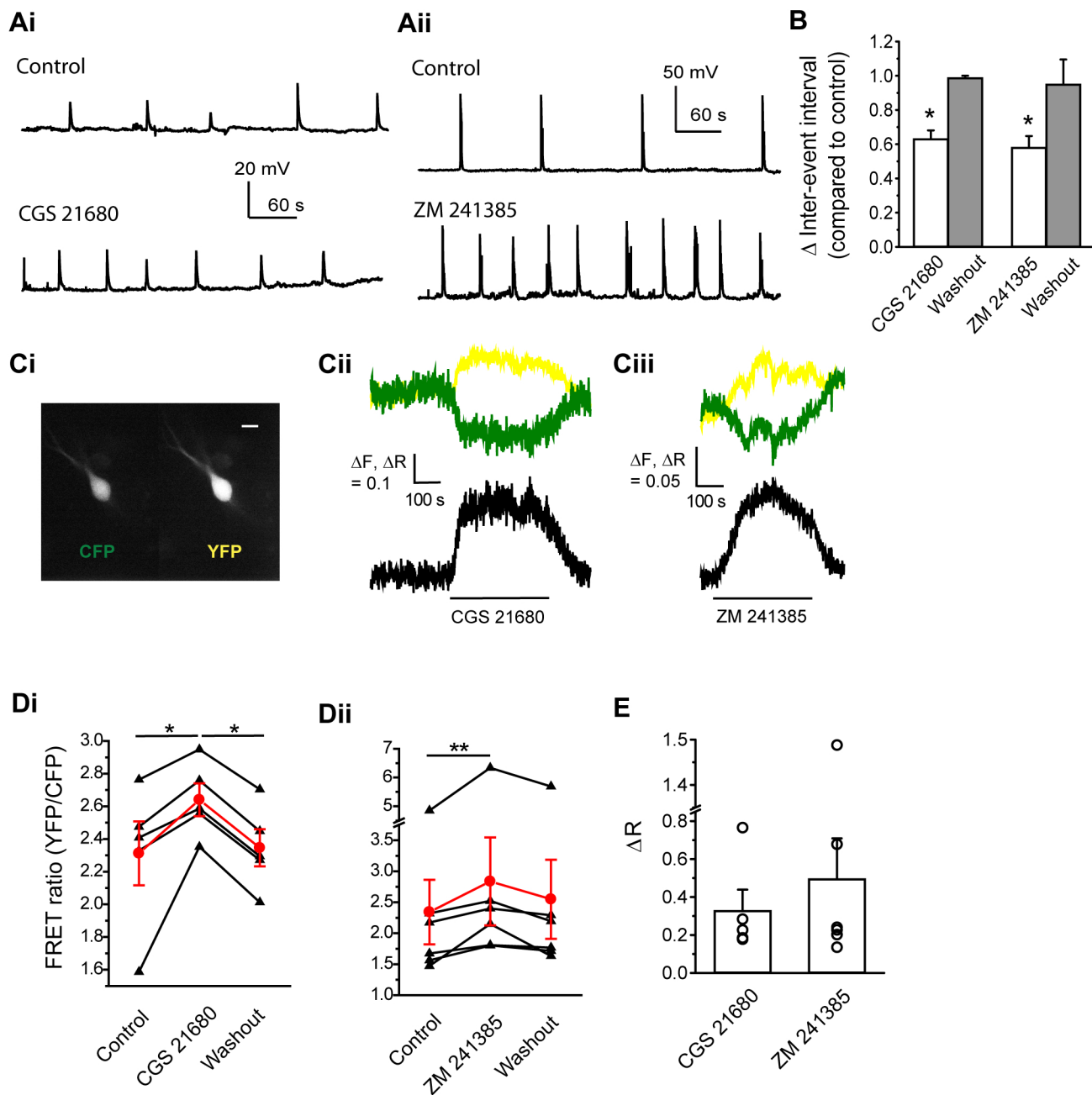

Figure S3

Supplement: Figure S3 — Both wave frequency and PKA activity are increased by the selective A2AR agonist or antagonist. (A) Wave-associated depolarizations in the absence (Control) and presence of A2AR agonist (Ai, 5 µM CGS 21680) or antagonist (Aii, 10 µM ZM 241385). (B) The average changes in the inter-event interval of wave-associated depolarizations in the presence or absence of the A2AR agonist or antagonist. Data were normalized to the control. Data were obtained from 4–6 acutely isolated P2 rat retinas. * p<0.05; Mann-Whitney method for comparing the presence and absence of the A2AR agonist; * p<0.05; two-tailed Student's unpaired t-test for comparing the presence and absence of the A2AR antagonist. (C) Ci, a cell expressing the FRET-based PKA activity reporter in the CFP or YFP channel [44], [45], [46], [47], [48], [49], [50], [51], [52]. Scale bar for both channels, 10 µm. Cii and Ciii, changes in the fluorescence intensity (ΔF) (green traces, acquired from the CFP channel; yellow traces, acquired from the YFP channel); changes in the FRET ratios (ΔR) (black traces) [13], [44], [45], [46], [47], [48], [49], [53], [54] upon application of an A2AR agonist or antagonist. (D) The FRET ratios before, during and after the application of an A2AR agonist (Di) or antagonist (Dii). Black, FRET ratios from individual cells. Red, average FRET ratios from cells. (Di) *p<0.05, repeated measures ANOVA; (Dii) **p<0.01, Friedman test. (E) The average changes in the FRET ratios (ΔR) induced by an A2AR agonist or antagonist. Each circle indicates the experiment from one cell. Data were obtained from 5–6 cells, 3 transfected retinas, and 3 pups. p = 0.51; two-tailed Student's unpaired t-test. (PDF) [file pone.0095090.s003.pdf]
