# Supplementary material for: Adenosine A2A Receptor Up-Regulates Retinal Wave Frequency via Starburst Amacrine Cells in the Developing Rat Retina
Source: PLoS One. 2014 Apr 28;9(4):e95090. doi: 10.1371/journal.pone.0095090 (PMC4002430; doi:10.1371/journal.pone.0095090)
Supplement: Table S1 — Ca2+ transient characteristics following expression of A2AR under the control of the CMV promoter. (PDF) [file pone.0095090.s004.pdf]

Table. S1. Ca^{2+} transient characteristics following expression of $A_{2A}R$ under the control of the CMV promoter.

	Inter-wave interval (sec)	*Duration (sec)*	*Amplitude (% $\Delta F/F$)*	*# Retinas*
**Control**	94.34 ± 15.20	16.84 ± 2.42	2.02 ± 0.65	6
**pCMV-$A_{2A}R$**	109.54 ± 12.84	16.98 ± 2.58	2.15 ± 0.54	9

Ca^{2+} transient characteristics were measured from cultured whole-mount retinas expressing control vector (pCMV-IRES2EGFP) or wild-type $A_{2A}R$ driven by the CMV promoter (pCMV-A_{2A}R-IRES2EGFP). No significant differences were found between control and pCMV-A_{2A}R. For inter-wave interval, $p = 0.46$; two-tailed Student's unpaired *t*-test. For Ca^{2+} transient duration, $p = 0.97$; two-tailed Student's unpaired *t*-test. For Ca^{2+} transient amplitude, $p = 0.99$; Mann-Whitney method.
