# Supplementary material for: Adenosine A2A Receptor Up-Regulates Retinal Wave Frequency via Starburst Amacrine Cells in the Developing Rat Retina
Source: PLoS One. 2014 Apr 28;9(4):e95090. doi: 10.1371/journal.pone.0095090 (PMC4002430; doi:10.1371/journal.pone.0095090)
Supplement: Text S1 — Methods: Fluorescence resonance energy transfer (FRET) imaging and Pharmacology. (PDF) [file pone.0095090.s005.pdf]

Methods

Fluorescence Resonance Energy Transfer (FRET) imaging

To monitor the PKA activity in the developing rat retina, we transfected P2 rat retinas with the genetically encoded fluorescence-based reporter, A-kinase activity reporter (AKAR3), consisting of the fusion of cyan fluorescent protein (CFP), a phosphothreonine-binding domain (FHA1), a PKA consensus phosphorylation target, and the yellow fluorescent protein (YFP) variant citrine [44,45,46,47,48,49,50,51,52]. In response to phosphorylation by PKA, this PKA activity reporter undergoes conformational changes to bring the CFP and the YFP variant in a close position; thus, the ratios of fluorescence resonance energy transfer (FRET) were increased [13,44,45,46,47,48,49,53,54] (Figure S3-Cii-Ciii).

Live FRET imaging was performed on an upright fluorescent microscope (Olympus BX51WI) using a 60× water immersion objective (Olympus LUMPLFLW). During FRET imaging, the retinal explants expressing AKAR3 were continuously superfused with oxygenated ACSF at 30°C. To acquire the FRET signal, CFP was excited by a narrow bandwidth (436/10 nm). Two emission wavelengths were collected simultaneously using a Dual-View image splitter (Optical Insights) with appropriate YFP (535/30 nm) and CFP (480/40 nm) emission filters. Images were captured with a CCD camera (CoolSNAP HQ2, Photometrics) at 2 s-intervals, with exposure times ranging from 250 to 500 ms. Digitized imaging data were subsequently analyzed by MetaMorph, with background subtraction from both channels and the correction of CFP bleed-through into the YFP channel according to the previous study [13].

FRET ratios were calculated as $R = (F_{YFP}/F_{CFP})$ for individual cells (Figure S3-Cii-Ciii). Before, during and after drug application, the values of the FRET ratio were calculated by averaging over five images around the maximum response (Figure S3-Di-Dii). The magnitude of drug-induced change was reported as the change in FRET ratios (ΔR), computed by subtracting the value of the FRET ratio before drug application from the value of the FRET ratio around the maximum response to drug (Figure S3-E).

Pharmacology

The selective $A_{2A}R$ agonist (CGS 21680) or antagonist (ZM 241385) and the PKA inhibitor (H89) were purchased from Tocris Biosciences and prepared as 1000× concentrated stock solutions in Dimethyl sulfoxide (DMSO). Stocks were stored at -20°C and diluted in ACSF on the day of the experiments. Drug application was delivered by a perfusion system.
